# Allergic Conjunctivitis: Review of Current Types, Treatments, and Trends

**DOI:** 10.3390/life14060650

**Published:** 2024-05-21

**Authors:** Fiza Tariq

**Affiliations:** Pennsylvania College of Optometry, Salus University, Elkins Park, PA 19027, USA; ftariq@salus.edu; Tel.: +1-215-421-2508

**Keywords:** allergic conjunctivitis, ocular allergy, IgE antibodies, Th2 lymphocytes, antihistamines, mast cell stabilizers

## Abstract

Allergic conjunctivitis is an allergen-induced immune response secondary to the binding of immunoglobulin-E (IgE) to sensitized mast cells. Approximately 40% of North Americans and 20% of the world’s population are impacted by some form of allergy and it continues to increase in prevalence, especially among children. Specified IgE antibodies can be found in almost all cases of exposure to seasonal or perennial allergens. Activation and degranulation of mast cells lead to increased tear levels of histamine, tryptase, leukotrienes, cytokines, and prostaglandins. The release of these factors initiates the recruitment of inflammatory cells in the conjunctival mucosa, which causes the late-phase reaction. Signs and symptoms of ocular allergies include itching, tearing, chemosis, and hyperemia, which can lead to decreased productivity and poor quality of life. Many treatment options are available to improve symptoms, including, mast cell stabilizers, antihistamines, dual-acting agents, steroids, nonsteroidal anti-inflammatory drugs (NSAIDS), and other off-label treatment modalities. This review article provides an overview of different types of allergic conjunctivitis, its pathology and immunology, and recommended methods of treatment.

## 1. Introduction

Allergic eye disease is an ocular manifestation of the body’s immune response to other normally harmless substances known as allergens. Approximately 40% of North Americans and 20% of the world’s population are impacted by some form of allergy, making it one of the most commonly encountered clinical conditions [1,2,3]. Allergic conjunctivitis can result from various impacting factors including genetics, air pollution, atopy, pollen exposure, inflammation, and pet hair [3]. Examples of common allergens to the conjunctival surface include tree/grass pollen, house dust mites, animal/pest dander, and mold spores [3,4].

The classification of allergic conjunctivitis was revised a few years ago by the European Academy of Allergy and Clinical Immunology (EAACI), which details two types of ocular surface hypersensitivity disorders: ocular allergy and ocular non-allergic hypersensitivity [2].

Ocular allergy can be caused by IgE or non-IgE-mediated mechanisms. The first two most common and milder types of IgE-mediated ocular allergy include seasonal allergic conjunctivitis (SAC) and perennial allergic conjunctivitis (PAC). The two more severe and chronic forms of IgE-mediated mechanisms include vernal keratoconjunctivitis (VKC) and atopic keratoconjunctivitis (AKC). Non-IgE-mediated forms of ocular allergy are less common and include contact blepharoconjunctivitis (CBC), VKC, and AKC. The second type, ocular non-allergic hypersensitivity, includes giant papillary conjunctivitis (GPC), irritative conjunctivitis, irritative blepharitis, and other mixed forms [2].

SAC is the most common form of ocular allergy and accounts for approximately 90% of all cases, with PAC as the second most common [1,2]. In addition to being the most common, they are also the milder forms of ocular allergies behind VKC and AKC. Despite its high prevalence and common presentation, allergic eye disease is often an underdiagnosed and undertreated health problem. The presenting symptoms of itching, redness, and swelling may seem mild but can significantly impact a person’s daily quality of life [2,3]. About 10% of patients with ocular allergy symptoms usually tend to self-medicate and/or fail to seek medical attention. The predominance of self-management increases the risk of suboptimal treatment, leading to recurring symptoms and worsening chronic exacerbations that can impact the cornea and vision [5]. Multiple overlapping conditions, such as infectious diseases and dry eye syndromes, make it challenging to arrive at the correct diagnosis/diagnoses [2,5]. Therefore, successful management includes thorough patient history and appropriate ophthalmological techniques for diagnosing and providing accurate treatment options [1,2,5].

In this comprehensive review paper, I thoroughly assess the peer-reviewed literature on the latest trends, current types, and treatments available for allergic conjunctivitis. The databases and libraries used for the article search include PubMed, Science Direct Journals, and OVID Journals. The search criteria date ranges were restricted to 2004–2024 to ensure that the most recent data were accessed. The keywords in the search included ocular allergy, allergic conjunctivitis, IgE antibodies, Th2 lymphocytes, antihistamines, and mast cell stabilizers. My inclusion criteria included well-written original yet comprehensive articles, including clinical research, book chapters, and reviews emphasizing the immunological basis of ocular allergy. My exclusion criteria included studies lacking a pathological, physiological, or immunological understanding/basis, studies missing methodological rigor, studies not written in English, and poor organization. This thorough methodological process ensured a scientifically sound synthesis of our present-day understanding of allergic conjunctivitis. This article highlights the current trends, types, and treatments of allergic conjunctivitis from a pathophysiology and immunological perspective.

## 2. Etiology

Most cases of allergic conjunctivitis occur simply due to exposure to allergens on the ocular surface. Specifically, SAC, also known as hay fever conjunctivitis, is an acute disease that tends to worsen during the spring and summer seasons, and the most common allergens responsible are tree and grass pollen. On the other hand, PAC is chronic with remission and exacerbation periods and is present throughout the year [2,3]. The difference between the two is rooted in the allergen types: SAC occurs typically due to outdoor airborne allergens that are worse in the spring, and PAC occurs due to indoor airborne allergens throughout the year [2,3]. The exact cause of VKC is unknown but it is correlated with certain climate and environmental exposures. VKC can be classified into three different forms based on its clinical presentation: palpebral, limbal, and mixed [2,6]:

- Palpebral VKC largely affects the upper tarsal conjunctiva and significantly involves the cornea and its damage from the overlying inflamed conjunctiva.
- Limbal VKC predominately affects individuals of Black and Asian descent and primarily manifests in temperate climates.
- Mixed VKC exhibits a combination of features seen in both palpebral and limbal disease, including involving the upper tarsal conjunctiva and the limbal area.

The exact etiology of AKC remains unclear but it has been connected to various factors, including genetic predisposition and atopic dermatitis (present in more than 90% of cases). GPC can be correlated to ocular foreign bodies that can either carry allergens or cause damage to the ocular surface, leading to easier allergen infiltration [3]. This condition can be associated with various ocular foreign bodies such as contact lenses, ocular prostheses, exposed scleral buckles, glaucoma filtering blebs, and sutures, amongst others [7].

## 3. Pathophysiology

The ocular surface consists of the cornea and the conjunctival mucosal barrier, which protects the eye from foreign invasion and is a common site of allergic inflammation due to its easy access to airborne allergens [8]. The ocular surface is blanketed by the tear film, which consists of a lipid, aqueous, and mucin layer formed by the meibomian glands, lacrimal glands, and goblet cells, respectively [9,10]. The tear film plays a vital role in visual acuity. It lubricates and protects the epithelium of the ocular surface [11]. The ocular surface is an “immune-privileged” site as it maintains corneal transparency and integrity by suppressing unnecessary inflammatory responses while maintaining the capability to mount an effective immune response against pathogens [8,12,13]. The cornea is avascular and has no lymphatic drainage; thus, no active blood-circulating leukocytes can enter or collect in the corneal tissue [12,14]. The healthy cornea also does not have any mature leukocytes, which decreases its ability to produce pro-inflammatory cytokines and are limited in their ability to produce lymphoid cells [15,16]. The cornea is kept clear by producing anti-inflammatory cells such as regulatory T cells, IgA-producing plasma cells, and immunosuppressive cytokines [17,18,19]. 

Unlike the cornea, the conjunctiva harbors a diverse group of immune cells (primarily T cells) during its steady state. The conjunctival epithelial consists of goblet cells, CD8^+^T cells, and Langerhans cells, and the subepithelial layer of the conjunctiva consists of blood vessels, lymphatics, macrophages, dendritic cells, fibroblasts, and mast cells [17,18,19]. This distribution of immune cells in the conjunctival mucosa is known as the conjunctival-associated lymphoid tissue (CALT) [18,20]. The CALT consists of conjunctival lymphoid follicles (CLFs) and diffuse lymphoid effector tissue. CLFs consist of B cells and T cells, whereas diffuse lymphoid effector tissue consists of mast cells, macrophages, IgA-secreting plasma cells, and effector T cells [20,21,22,23].

The major effector cell responsible for the majority of allergic inflammation responses is the mast cell [11,17]. In the acute phase, the cross-linking of IgE on the surface of mast cells releases preformed mediators of histamine, tryptases and leukotrienes, which play a major role in the clinical symptoms associated with allergy [24]. The late phase is characterized by the release of various chemokines and inflammatory proteins and the infiltration of eosinophils, basophils, T cells, neutrophils, and macrophages that lead to further conjunctival inflammation [11,25]. 

The conjunctival epithelial has tight junctions that prevent allergens from gaining access to the subepithelial layer [26,27]. In allergic conjunctivitis, this barrier function is compromised due to the activation of the protease-activating receptor [26,28]. This leads to the release of cytokines, chemokines, and adhesion molecules as part of the allergen-included immune response [22]. These mediators are released by the conjunctival epithelial cells and encourage the influx of more immune cells to the site of inflammation [26,28].

The immunopathogenic mechanisms of SAC and PAC are usually type-1 hypersensitivity, IgE-mediated responses involving mast cells, whereas, in chronic allergic disorders like VKC or AKC, the mechanisms are complex, including both IgE- and T-cell-mediated responses. The immunological response to ocular allergy can be broken down into three phases: sensitization, the early/acute phase, and the late/chronic phase. 

### 3.1. Sensitization

The first phase of the IgE-mediated immune response is sensitization [29]. This phase defines the initial exposure of the allergen to the conjunctival mucosa. Once an allergen is deposited on the conjunctiva, it is processed and cleaved into peptide fragments by the Langerhans cells, dendritic cells, and other antigen-presenting cells (APCs) on the mucosal epithelium [29,30]. These peptide fragments are displayed on the surface of the APCs by major histocompatibility complex (MHC) class II molecules [30]. The peptide/MHC II complex interacts with the T-cell receptor (TCR) on naïve CD4^+^T-lymphocytes. In conjunction with other co-stimulatory molecules, the MCH-TCR interaction activates the CD4^+^T-lymphocytes into T Helper type 2 (Th2) lymphocytes [29,30]. The Th2 lymphocytes then interact with B-lymphocytes, which trigger the release of Th2-lymphocyte-mediated cytokines (IL-3, IL-4, IL-5, IL-6, IL-10, and IL-13) [30]. The release of IL-4 and other accessory molecules leads to the conversion of B-lymphocytes into antibody-producing plasma cells. These plasma cells undergo immunoglobulin class switching, which leads to the production of antigen-specific IgE. These IgE antibodies are now specific to the initial allergen and prime mast cells and basophils by binding to their surface receptors. These cells are now ready for subsequent exposure to allergens and mark the completion of the process of sensitization [29,30].

### 3.2. Early Phase

Once an allergen is re-presented to sensitized mast cells, an allergic reaction is initiated. The natural environmental allergen binds to the IgE molecules on the mast cell receptors, leading to the cross-linking of molecules and subsequently signaling the degranulation of mast cells [29,30,31]. The activation and degranulation of mast cells lead to a cascade of events, including the activation of the arachnoid acid pathway, the release of chemokines (such as eotaxin), and the release of preformed mediators such as histamine and tryptase. This marks the initiation of the early phase of allergic conjunctivitis. The increased levels of histamine cause the dilation of blood vessels, the stimulation of nerve endings, and the activation of mucous-producing cells, leading to the clinical manifestations of an ocular allergic reaction [30,31]. The presenting signs and symptoms include itching (a hallmark sign of ocular allergy), hyperemia, chemosis, tearing, mucus discharge, and eyelid swelling [2,3,29,30,31]. The mast cell degranulation also leads to the activation of vascular endothelial cells and the release of adhesion molecules, chemokines, and cytokines [29,30,31]. This also leads to the biosynthesis of lipid mediators such as prostaglandins and leukotrienes, resulting in the recruitment of inflammatory cells into the conjunctival mucosa and initiating the late phase of the allergic reaction [30].

### 3.3. Late Phase

**Figure 1:**
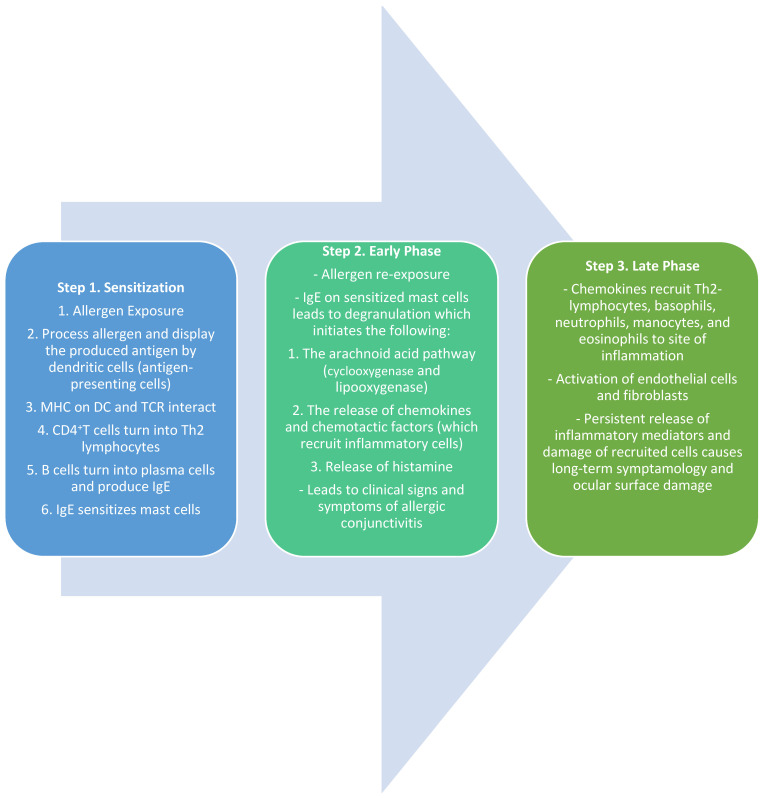
Summary of the sensitization and early and late phases of ocular allergy [7,8,9].

The late phase of the allergic response typically occurs about 6–12 h after the initial exposure. The release of chemokine factors from the early phase is responsible for the recruitment and infiltration of eosinophils, basophils, neutrophils, Th2 lymphocytes, and monocytes into the conjunctiva [29,30]. This phase also includes the maturation of Th2 lymphocytes and the production and release of Th2-mediated cytokines such as IL-4, IL-5, and IL-13. IL-4 and IL-13 play an active role in the formation of giant papillae by stimulating conjunctival fibroblasts and overexpressing IgE [29,30,32,33]. IL-5 recruits and activates eosinophils which leads to continued inflammation, persistent symptoms, and an increased likelihood of long-term tissue damage, as seen in VKC [30,32] (Figure 1).

## 4. Seasonal/Perennial Allergic Conjunctivitis (SAC/PAC)

The most prevalent form of allergic conjunctivitis is attributed to seasonal and perennial conjunctivitis. The difference is mostly based on the periodicity and chronicity of the symptoms and the type of allergens. SAC symptoms are frequently caused by transient allergens such as tree and grass pollen and usually occur in the spring and summer seasons [2,32,34]. SAC is frequently associated with allergic rhinitis (hay fever) and asthma [32,35]. On the other hand, perennial allergic conjunctivitis (PAC) symptoms are typically caused by indoor allergens such as house mites, dust, pet dander, and mold spores and cause symptoms persistently throughout the year with periods of remission and exacerbation [2,32,34]. Many patients can also be polysensitized in that they experience perennial allergic symptoms with seasonal exacerbations [2]. Clinical presenting signs and symptoms are usually bilateral and include itching (main symptom), hyperemia, tearing, conjunctival papillae, and chemosis [2,32,34]. Conjunctival hyperemia is usually mild to moderate in presentation, whereas conjunctival chemosis is moderate to severe and corneal involvement is rarely present. In severe cases, symptoms of blurred vision and photophobia can also be present [34]. Patients can also present without any symptoms at the visit; thus, it is important to obtain a thorough history including symptoms around different times of the year, systemic conditions, and current medications.

## 5. Vernal Keratoconjunctivitis (VKC)

VKC is a rare, bilateral, chronic, and severe form of allergic eye disease that can permanently cause vision loss if left untreated [34]. It is more commonly present in young, prepubescent males with a high incidence between 11 and 13 years of age [35,36]. The word vernal means “spring”, as VKC typically presents with exacerbated symptoms during hot spring–summer seasons [36,37]. The condition is more prevalent in the continents of Asia, Africa, and South America where warm, dry, and windy climates are common [38]. Studies indicate that nearly 50% of patients with VKC have atopic sensitization and a correlation with atopic-associated conditions such as asthma, rhinitis, and eczema [29,30,37,39]. 

VKC is recognized due to its chronic allergic inflammation secondary to the activation of Th2 lymphocytes and mediated cytokines [37,38]. The activated Th2 lymphocytes initiate a cascade of cellular mechanisms that are responsible for the overexpression of IgE and the recruitment and activation of eosinophils and mast cells through pro-inflammatory cytokines (IL-4, IL-5, IL-13) [29,30]. Pathological examination of the tears of VKC patients indicates increased levels of tumor necrosis factor (TNF-alpha), histamine, tryptase, and IgG and IgE [30,38,39]. Th2 lymphocytes, mast cells, eosinophils, and their correlated mediators play a key role in the ocular manifestation of VKC [30,38]. 

VKC typically presents with intense ocular symptoms including itching, tearing, hyperemia, mucous/serous discharge, blurry vision, and photophobia associated with corneal involvement [37,38]. The defining presenting signs of VKC are giant conjunctival papillae of the upper tarsus and superior limbal region [37,39]. Another classic sign is transient limbal deposits full of eosinophils and epithelium debris, better known as Horner–Trantas Dots [38,39]. Repetitive, chronic eyelid–corneal interaction and cell-mediated inflammation can lead to corneal complications such as punctate epithelial erosions (PEEs), neovascularization, infectious keratitis, and corneal scarring [39]. Chronic and untreated superficial keratopathies can extend deeper into Bowman’s layer and cause shield ulcers, usually in the central or superior parts of the cornea [38,39]. Shield ulcers are a sight-threatening condition found in 3–20% of VKC patients and can lead to permanent vision loss [37,39]. Other complications correlated with severe VKC include dry eye disease, keratoconus, and limbal stem cell deficiency [38,39]. 

## 6. Atopic Keratoconjunctivitis (AKC)

AKC is a bilateral, chronic, inflammatory condition of the ocular surface [32,40]. Most patients present with accompanying systemic conditions such as atopic dermatitis and eczema [40,41]. Signs of blepharitis and scurf are also commonly found on the lashes with severe corneal findings such as neovascularization, scarring, and thinning [41]. It frequently presents in late adolescence with peak presentation between the ages of 30 and 50 [40,41]. The immunopathology of AKC is like other allergic conjunctivitis types as it is an IgE-mediated innate and Th2-lymphocyte-mediated inflammation of the eyelids and ocular surface. Pathological findings indicate an increase in IgE, TNF-alpha, eosinophils, mast cells, basophils, B cells, and T cells in the tears and conjunctiva [29].

Common presenting symptoms of AKC include typical pruritis, hyperemia, ropy mucoid discharge, burning, and tearing. However, its distinguishing feature is thickened, exudative, and eczematous eyelid lesions [39,41]. The eyelids also have a rough, sand-like appearance, and scratching makes them itchier [39]. Many AKC patients also have associated meibomian gland dysfunction and chronic blepharitis [39,40]. Other corneal complications include pannus, neovascularization, punctate erosions, and ulcerations, which can lead to corneal scarring and eventually permanent visual impairment [41]. If left untreated or overtreated with steroids, these patients can develop keratoconus, glaucoma, anterior and posterior subcapsular cataracts, and herpetic ocular disease [40,41].

## 7. Giant Papillary Conjunctivitis (GPC)

GPC is an inflammatory, non-infectious disease characterized by papillary hypertrophy of the superior tarsal conjunctiva [7,42]. Even though there is papillary involvement, GPC is not truly categorized as an allergic disease but rather presents when the superior palpebral conjunctiva becomes in chronic contact with a foreign body [7,29]. Even though it mostly occurs in conjunction with contact lens usage, it can occur because of an ocular prosthesis, exposed suture, glaucoma filtering blebs, exposed scleral buckles, and many others [7,32,34]. Patients typically present with symptoms like an allergic etiology presentation including mucous discharge, tearing, itching, and blurry vision. A key distinguishing factor, though, is complaints of diminished contact lens tolerance and increased lens awareness. It is also commonly termed contact lens papillary conjunctivitis with papillae more than 1mm in size [7]. 

The pathophysiology of GPC is multifactorial and is likely initiated with mechanical damage to the palpebral conjunctiva, leading to innate and adaptive immunity responses. It is hypothesized that the proteinaceous debris on the anterior surface of the contact lens acts as a presenting antigen to an IgE-bound mast cell on the conjunctival epithelium. Pathological testing indicates that these patients have high amounts of IgE and Ig-G and increased amounts of IL-3 and IL-4. Involved cell mediators include mast cells, basophils, Th2 lymphocytes, and the Th2-derived cytokines that perpetuate the inflammation process [7]. 

## 8. Contact Dermatoconjunctivitis (CDC)

CDC is a type IV delayed hypersensitivity reaction that impacts the eyelids and conjunctiva [34]. Presenting symptoms typically include pruritis, hyperemia, papillary/follicular reaction of the inferior palpebral conjunctiva, punctate keratitis, and dermatitis of the surrounding periocular skin [30,34]. The reaction is a Th1-lymphocyte-mediated reaction in which Langerhans cells process and present environmental allergens to T helper cells in the regional lymph nodes [34]. The Th1-lymphocytes, in turn, release cytokines and chemokines, activating and immigrating more inflammatory cells in the affected region [30,34]. Common allergens include mydriatic drugs, antibiotics, antiviral agents, glaucoma drops, anesthetics, preservatives, and cosmetics. In sensitized individuals, the immune response can take up to 48–72 h to develop, in contrast to toxic or irritant allergens that can induce an innate immune response within 2–3 h [34].

## 9. Treatment

The management of allergic conjunctivitis includes preventative measures as well as non-pharmacological and pharmacological treatment. The most effective treatment option for complete prevention of symptoms is avoiding the allergen to prevent triggering the initial cascade response [43,44,45]. However, complete avoidance is not always possible and requires identifying the offending agent. Recommendations can be provided to create an environment where allergen exposure is reduced. During the symptomatic period, preventative measures against airborne allergens include keeping windows closed, using screen filters, avoiding eye rubbing, wearing sunglasses, washing hands after being outdoors, and increasing patient awareness of monitoring seasonal pollen counts to avoid contact [1,43,44,46]. Allergens such as dust mites can be reduced by regularly washing/replacing bed covers, vacuuming the entire house at least weekly, decreasing humidity, and removing/regularly cleaning any areas that particularly gather dust (carpets, curtains etc.). Animal dander can be reduced by keeping animals outside, avoiding touching them or rubbing one’s eyes after exposure, and washing hands/clothes after coming in contact [43,44]. Other non-pharmacological interventions include using cool compresses to decrease edema and hyperemia in irritated eyes. Artificial tears can also be recommended to dilute and flush out allergens from the tear film and treat if they have any co-morbid disease [46,47].

Despite many options available for avoidance or non-pharmacological intervention, symptoms can persist, and the use of anti-allergy medication is needed to alleviate symptoms. With increased knowledge of the mechanism of action of ocular allergy, there has been an increase in the number of available anti-allergic medications that target the tissues and cells involved in an immunological response. A combination of topical and oral treatment options may be utilized to address first-line treatment options for allergies. Anti-allergic medications include topical vasoconstrictors, mast cell stabilizers, antihistamines, and dual-action agents combining mast cell stabilizing with antihistamine properties (Table 1). 

### 9.1. Topical Vasoconstrictors (Decongestants)

Topical vasoconstrictors (decongestants) were the first ocular medication approved for the treatment of allergic conjunctivitis. Over-the-counter (OTC) topical vasoconstrictors are effective at temporarily decreasing conjunctival hyperemia by stimulating alpha-adrenergic receptors [48]. Alpha-adrenergic agonists cause vasoconstriction of conjunctival blood vessels, resulting in decongestion and whitening of the eye [34,48]. However, the use of these agonists can lead to side effects such as rebound hyperemia and tachyphylaxis and, chronically, can lead to conjunctivitis medicamentosa [32,48]. Commonly used topical vasoconstrictors are oxymetazoline, naphazoline, tetrahydrozoline, and phenylephrine. These are best utilized as short-term solutions and should be avoided in narrow-angle glaucoma and cardiovascular issues [34,48]. These should not be recommended as a standalone treatment and used in combination with antihistamines for the treatment of allergic conjunctivitis [1,34].

### 9.2. Mast Cell Stabilizers

Mast cell stabilizers work by preventing the degranulation of sensitized mast cells, thus stopping the release of histamine and other inflammatory mediators [43,49]. Since mast cell stabilizers act before the mast cell is degranulated, they rarely have an impact on the inflammatory mediators once they are already released [48,49]. In other words, mast cell stabilizers are not effective once the patient is symptomatic, and clinical trials have had a difficult time showing their efficacy [48]. Since there are other quicker and more effective treatment agents available on the market, mast cell stabilizers are rarely used as monotherapy. The most common mast cell stabilizers used for allergic conjunctivitis are sodium lodoxamide 0.1% (Alomide), cromoglycate 2%, and nedocromil 2% [43,45]. Mast cell stabilizers can be utilized as a prophylactic measure to prevent mast cell degranulation for repeated exposures to the allergen [32,48].

### 9.3. Antihistamines

Antihistamines are competitive antagonists of histamine receptors that are present in the conjunctiva and eyelids. Once stimulated, these receptors lead to capillary dilation and increased vascular permeability, which leads to common allergic symptoms of itching and edema. Thus, antihistamines work by preventing the binding of histamine to H1 receptors and preventing the cascade of inflammatory events. Ocularly, only H1 receptors are available [50].

Oral antihistamines are easily accessible and a great add-on therapy in addition to topical allergy medications. First-generation antihistamines such as Diphenhydramine (Benadryl) are avoided due to their ability to cross the blood–brain barrier and produce unwanted side effects such as sleepiness, confusion, urinary retention, and worsening dryness [50,51]. On the other hand, second-generation antihistamines are much more desirable as they do not cross the blood–brain barrier and produce fewer anticholinergic effects [51]. Examples of second-generation oral antihistamines include fexofenadine (Allegra), loratadine (Claritin), and cetirizine (Zyrtec), all of which are readily available OTC. Oral antihistamines can be utilized as an adjunct therapy, especially when non-ocular allergy symptoms such as rhinitis are present [48]. 

First-generation topical antihistamines, antazoline and pheniramine, are available OTC; however, they are poorly tolerated and have a limited potency and short duration of effects [51,52]. These are often combined with the vasoconstrictors naphazoline and tetrahydrozoline, more commonly known as Visine or Clear Eyes. Second-generation topical antihistamines, levocabastine and emedastine, have a longer duration of action (4 to 6 h) and, thus, comparatively decreased dosing compared to first-generation antihistamines [50,51]. These were the first antihistamines to impact both the early and later responses of the immune system. Even though the newer-generation antihistamines showed improvements, they were discontinued in the United States [48]. 

### 9.4. Dual-Acting Agents (Mast Cell Stabilizers/Antihistamines)

Dual-acting agents combine the properties of mast cell stabilizers and H1 receptor antagonists (antihistamines) to demonstrate great efficacy and safety when compared to placebo [52]. Examples of dual-acting agents include azelastine, epinastine, alcaftadine, bepotastine, ketotifen, and olopatadine [48,51]. Some dual-acting agents, such as epinastine, act on both H1 receptors and H2 receptors by reducing pruritis and vasodilation, respectively, while others, such as azelastine, also inhibit platelet-activating factor (PAF) activity and reduce the expression of interleukin adhesion molecule 1 (ICAM-1) [48,50]. These agents have been demonstrated to act quickly to reduce symptoms with a lasting effect because of their ability to inhibit the release of mediators and stop the recruitment of inflammatory cells [48,49]. 

Olopatadine 0.1% (Pataday Twice Daily Relief) was the first topical anti-allergy medication that was approved for twice-daily usage [43]. These agents are all preserved with a surfactant called benzalkonium chloride that may cause ocular surface toxicity [52]. These are now considered the first line of treatment for allergic eye disease and are the most common ophthalmic agents recommended by eye care practitioners and allergists [43]. Thus, these agents can be used prophylactically to prevent mast cell degranulation and acutely following the onset of symptoms [53].

Compared to placebo, olopatadine has been found to improve symptoms of eyelid edema, hyperemia, chemosis, pruritis, and overall quality of life. Multiple randomized control trials have compared ketotifen and olopatadine. One meta-analysis found improvement in symptoms of itching after 14 days in favor of olopatadine 0.1% when compared to ketotifen 0.025% [54]. Before 2020, olopatadine was only available as a prescription medication, and ketotifen, in the form of Zaditor or Alaway, was clinically commonly prescribed as the first line of relief as an OTC medication. Within the last 4 years, olopatadine became available OTC and has gained popularity to become clinically superior to ketotifen in terms of efficacy. 

### 9.5. Topical NSAIDs

Topical nonsteroidal anti-inflammatory drugs (NSAIDS) act by blocking the cyclooxygenase enzymes (COX-1 and COX-2) within the cyclooxygenase pathway, resulting in the inhibition of inflammatory mediators such as prostaglandins and leukotrienes [48,49]. These drugs have proven efficacy against conjunctival hyperemia, pruritus, pain, and irritation [48]. Topical NSAID agents commonly associated with the relief of ocular allergy symptoms include ketorolac, diclofenac, indomethacin, and flurbiprofen [48,49]. Although ketorolac has been approved for treating allergic conjunctivitis, studies have indicated that it is less effective compared to topical antihistamine agents [48,49,55]. Moreover, these agents can cause burning and stinging sensations upon instillation, so long-term compliance is an issue. Thus, NSAIDs may be used for temporary relief of itching and hyperemia compared to no treatment; however, they do not aid with symptoms of mucous discharge, chemosis, and corneal damage, so alternative methods should be considered [48,49,55,56]. 

### 9.6. Corticosteroids

Glucocorticoids can be an effective form of treatment against more severe and chronic forms of allergic conjunctivitis as they are well-known to be fast and effective anti-inflammatory agents [49,51,57]. This efficacy is the result of a variety of effects on the allergy cascade, including delaying/inhibiting the release of inflammatory mediators to suppress the late-phase immunological response [49,57]. Specifically, this class of medications interferes with protein synthesis and stops phospholipase A2, the enzyme responsible for arachnoid acid, and inhibits the production of leukotrienes and prostaglandins [48,49,58]. Steroids also impact other aspects of the immunological response including inhibiting the proliferation of mast cells, decreasing the production of eosinophils, and reducing the availability of histamine [59,60]. With these tremendous anti-inflammatory effects comes the cost of ocular adverse effects. Corticosteroid side effects include delayed wound healing, cataract formation, elevated intraocular pressure, and superinfections, indicating the need for close monitoring [34,49,58,59,60]. Ketone-based medications such as prednisolone and dexamethasone are highly potent with a high efficacy; however, they are also accompanied by a large likelihood of steroid-induced ocular complications [31,48,59]. On the other hand, ester-based or “soft” steroids are preferred for the treatment of moderate inflammation in allergic conjunctivitis, as they are more easily metabolized and carry fewer side effects [31,60,61]. Loteprednol etabonate 0.2% (which is approved for SAC) or fluorometholone (FML) 0.1% may be used on a short-term basis and in adjunct with mast cell stabilizers to combat signs of acute inflammation due to allergies [61,62]. Even with the milder form, close monitoring and caution should be exercised with any steroids to avoid long-term side effects [62].

### 9.7. Immunomodulators/Immunotherapy

Immunomodulatory agents are a nonsteroidal alternative to the therapeutic management of allergic conjunctivitis. They inhibit T-lymphocyte activation and proliferation, which prevents histamine release from mast cells and basophils, preventing chronic inflammatory damage to the ocular surface [29,31]. Both cyclosporin A and tacrolimus have been evaluated for safety and efficacy in more severe forms of allergic conjunctivitis including VKC and AKC and have been effective in reducing ocular signs and symptoms [63,64,65]. These drugs are calcineurin inhibitors, which allows them to be safe for long-term topical use without lasting side effects [29,31]. Allergen-specific immunotherapy is another highly effective treatment against severe allergic conjunctivitis/rhinoconjunctivitis and is recommended by the World Health Organization as an essential component of allergy management [49,52]. Both sublingual immunotherapy (SLIT) and subcutaneous immunotherapy (SCIT) seem to be effective routes of administration for treating nasal and ocular symptoms of severe allergy symptoms [48,52]. This mode of therapy works by introducing increasing doses of the allergen to a sensitized individual which would lead to desensitization and reduced allergic symptomology even after treatment cessation [51,52]. 

### 9.8. Contact Lenses

Typically, the treatment of allergic conjunctivitis includes temporary or permanent cessation of contact lens use [66]. However, patients may not be happy with the need to wear spectacles, and different contact lens types may be used to suppress/combat symptoms. When fitting a patient with a diagnosed ocular allergy, it is important to consider a daily disposable lens and avoid extended wear [67]. Moreover, the regular replacement of contact lenses and good contact lens hygiene is recommended to stop allergic exacerbations. Recent studies have also explored contact lenses as a therapeutic vehicle to release pharmaceuticals in a controlled dose, such as an antihistamine [68,69,70]. This could potentially increase patient compliance with topical treatments and allow controlled release throughout the day without needing to remember frequent dosing [71]. Another option is rigid gas-permeable scleral contact lenses that can shield the eye from mechanical trauma of the lids (especially in cases of GPC), prevent tear film evaporation, and provide a lubricated chamber for corneal protection and healing [72,73].

## 10. Conclusions

Allergic conjunctivitis is a highly prevalent ocular disease that continues to be underdiagnosed and undertreated. Its signs and symptoms can cause everyday discomfort and can significantly impair quality of life. Allergic conjunctivitis is largely a type-1 IgE-mediated hypersensitivity reaction where eosinophils, mast cells, and Th2 lymphocytes play a pivotal role in the sensitization and early and late phases of the immunological response. A thorough history and slit-lamp examination are key to correctly identify allergic eye disease and rule out any other underlying pathology. A wide range of non-pharmacological and pharmacological treatments are available that can be tailored to the needs of each patient. Eyecare specialists, primary care providers, and allergists each play an important role in patient education and management. This review article highlights the most up-to-date information regarding diagnosis, pathogenesis, and therapeutic options for various forms of allergic conjunctivitis. 

## Figures and Tables

**Table 1:** Summary of topical treatment of allergic conjunctivitis [31,48].

Drug Class	Mechanism of Action	Common Examples	Ocular Side Effects
Vasoconstrictors (Decongestants)	a-adrenergic agonists (mainly stimulation of a-1 receptors)	Phenylephrine, Brimonidine, Ephedrine, Naphazoline, Tetrahydrozoline	Rebound redness, conjunctivitis medicamentosa
Antihistamines	Competitive blockage of histamine receptors (all block H1 with some blocking H2, H3 and/or H4)	Levocabastine, Emedastine	Dryness, irritation
Mast Cell Stabilizers	Inhibit degranulation of mast cell and consequent histamine release	Sodium cromoglycate, Nedocromil sodium, Pemirolast, Lodoxamide	Stinging, Burning
Dual-Acting Agents	Inverse agonists of histamine receptors and prevent mast cell degranulation	Olopatadine, Ketotifen, Azelastine Epinastine, Alcaftadine	Burning, headache, dry eye
NSAIDS	Inhibits cyclooxygenase enzymes (COX-1 and COX-2) resulting in inhibition of prostaglandins	Ketorolac, Diclofenac Flurbiprofen	Stinging, burning, corneal melt
Corticosteroids	Inhibits phospholipase A resulting in the inhibition of prostaglandins and leukotriene synthesis	Dexamethasone, Prednisolone, Loteprednol, Fluorometholone, Rimexalone	Increased intraocular pressure, cataract formation, delayed wound healing
Immunomodulators	Cyclosporin A, Tacrolimus	Inhibiting production of IL-2 resulting in inhibition of T-cell activation	Burning, irritation
